# Single step route to highly transparent, conductive and hazy aluminium doped zinc oxide films†

**DOI:** 10.1039/c8ra09338e

**Published:** 2018-12-19

**Authors:** Jianwei Li, Sanjayan Sathasivam, Alaric Taylor, Claire J. Carmalt, Ivan P. Parkin

**Affiliations:** Materials Chemistry Centre, Department of Chemistry, University College London 20 Gordon Street London WC1H 0AJ UK i.p.parkin@ucl.ac.uk +44 (0)20 7679 7463; Department of Electronic & Electrical Engineering, University College London Torrington Place London WC1E 7JE UK

## Abstract

Light scattering yet transparent electrodes are important for photovoltaics as they increase device efficiency by prolonging light path lengths. Here, we present a novel single step route to highly textured Al doped ZnO thin films on glass substrates that show a minimum resistivity of ∼3 × 10^−3^ Ω cm and high visible light transmittance of 83% while still maintaining high haze factor of 63%. Roughness was imparted into the ZnO films during the synthetic process using acetylacetone and deionized water as additives. The highly hazy yet visible and near infrared transparent nature of the conductive ZnO:Al films allow it to be potentially used as an electrode material in amorphous and microcrystalline silicon solar cells.

## Abstract 2

A single step chemical vapour deposition route to highly conductive, transparent and hazy Al doped ZnO.

## Graphical abstract

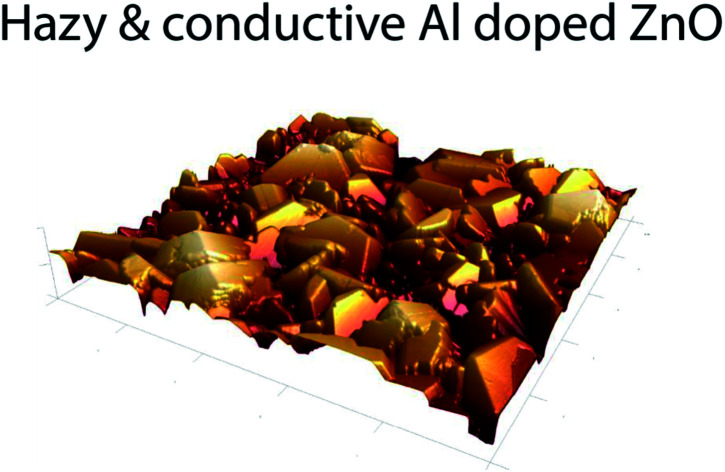

## Introduction

Transparent conducting oxides (TCOs) are a special class of materials that combine low electrical resistivity (<10^−3^ Ω cm) and visible light transparency (>80%) and are used as electrodes in photovoltaics and touch screen displays.^1^ Examples of widely used TCOs include Sn doped In_2_O_3_ (ITO), F doped SnO_2_ (FTO) and Al doped ZnO (AZO). Within these class of materials there exists specialized textured TCOs that perform not only as transmissive front electrodes and diffusion barriers but also as light trapping units. These units feature widespread light scattering sites at the interface and/or inside of the coatings that prolong light paths with various refractive indices.^2^ These are particularly important for silicon thin film solar cells.^3^ The limited absorption coefficient of amorphous (a-Si:H) and microcrystalline silicon (μc-Si:H) associated with their indirect band gap results in insufficient absorption of visible and the near infrared light (780 to 1100 nm) solar radiation during a single pass.^4^ Therefore, a layer of textured TCOs can substantially enhance the silicon absorber layer performance.^5,6^ Experimental work carried out by Sai *et al.* showed that in a p–i–n and n–i–p μc-Si:H device, having textured front and/or rear electrodes did result in a significant enhancement of light absorption in the visible and near infrared regions.^5^ Optical simulations have also shown that long wavelength quantum efficiency gains can be achieved in a-Si:H/μc-Si:H solar cells by using ‘W’ textured TCO substrates.^4^

Al doped ZnO has been extensively considered as a promising candidate for this application due to high resistivity to the hydrogen plasma that is used in the production of a- and μc-Si photovoltaics and ease of production at a large-scale from relatively inexpensive raw materials.^7^ However, arbitrarily generating a rough surface may not be able to offer good optical and electrical properties of TCO films and can occasionally even deteriorate mechanical property with an unexpected introduction of defects to the microstructure. Therefore, the majority of published research on textured ZnO:Al with high light scattering capabilities were carried out by two schemes: (i) a post treatment of as-prepared films by plasma/laser or wet chemical etching techniques for tailored rough surface morphology^8–11^ or (ii) using reactive ion etched rough glass as a substrate for film growth.^12–14^ Both of these treatments lead to increased capital production costs and can degrade the optoelectrical properties of the materials. A prerequisite for production of these materials and the widespread incorporation as a high-performance electrode is the identification of reliable, environment-friend and cost-effective synthetic routes to form scattering TCO materials free from parasitic absorbances.

There are numerous two-dimensional fabrication techniques for ZnO/AZO, which have been extensively investigated.^15–18^ However, demand for improved fabrication techniques which are facile and require less hazardous precursors remains high within the research community due to fierce market competition and a pressing need for environmental sustainability. Therefore, a robust design of aerosol-assisted chemical vapor deposition (AACVD) technique utilising the inexpensive precursors of zinc acetate dihydrate, deionized water, acetylacetone and aluminium trichloride dissolved in methanol was adopted in our studies for achieving high quality textured undoped ZnO and AZO with favorable optical properties, tailored towards Si-based thin-film solar-cell applications.

The AACVD technique has demonstrated facility for the formation of ZnO nanomaterials with precise microstructure control through the incorporation of either organic additives or dopants.^19–21^ For instance, McNally *et al.* first presented a series of modification of ZnO films by using various amounts of cationic surfactants, such as cetyltrimethylammonium bromide and tetraoctylammonium bromide, which gave rise to control of both crystalline orientation and morphologies by varying the concentration of the surfactants rather than the typical method of changing limited reaction parameters such as substrate temperature and solute concentration.^22^ Furthermore, Chen *et al.* also reported an AACVD route involving acetic acid as an additive to modify ZnO thin films, grown on textured FTO substrates. These films exhibited pyramidal shaped crystal grains interlaced together with a large grain size and different crystal structures compared with untreated ZnO samples.^23^ Additionally, it is known that dopants have a large influence on microstructures and functional properties of as-grown films in indium doped ZnO deposited by AACVD as reported Nolan *et al.*^24^ Therefore, the use of additives and dopants in AACVD system is highly effective to manipulate functional properties of ZnO thin films.

Herein, we present a novel single step synthetic route *via* AACVD by utilizing a combination of additives and dopants to generate highly textured ZnO:Al thin films processing both favorable electrical conductivity (minimum resistivity ∼3 × 10^−3^ Ω cm with a thickness of 780 nm) and optical properties (average transmittance and haze factor, inclusive of the silica coated float glass substrate, of 83.8% and 63% respectively across the visible spectrum including). This represents highly competitive optical performance and as an *in situ* microstructure forming technique, compares favorably with the commonly reported multi-step ZnO:Al fabrication routines,^25,26^ and hence has industrial applicability for photovoltaic cell production. Moreover, and rather surprisingly, we found that the addition of acetylacetone and D.I. water into the precursor solution resulted in our undoped ZnO thin films exhibiting a maximum haze factor of 78% in the visible range with a resistivity of 4.96 × 10^−2^ Ω cm associated with the high electron mobility of 22.9 cm^2^ V^−1^ s^−1^ and carrier concentration of 6.06 × 10^18^ cm^−3^ for a film thickness of 920 nm. The relatively high mobilities and high roughness are favorable for applications as electron transport layers in perovskite solar cells and transistors.^27,28^ Additionally, varying dopant concentrations were also examined in our work to reveal a deep insight into the interplay between additives and electrical/optical properties of the coating.

## Experimental

Zinc acetate dihydrate (>97%), acetylacetone (reagent grade, ≥98%) and aluminium trichloride (extra pure, anhydrous, granules, 99%) were purchased from Alfa Aesar™, Honeywell™ and ACROS™, respectively. Additionally, methanol was purchased from Fisher Scientific™ (HPLC grade) and nitrogen gas received from BOC™ (Surrey UK, 99.99%). Deionized water was taken from Elga DI water system and the standard float glass substrate with 50 nm thick SiO_2_ ion-diffusion inhibiting layer coated on the top surface was supplied by Pilkington NSG.

The typical laboratory AACVD set-up is illustrated in the literature,^29–34^ and preparation of aqueous solution for acetylacetone and D.I. water co-treated ZnO thin films was carried out by dissolving 0.4 g of zinc acetate dihydrate, 0.5 mL acetylacetone and 0.5 mL D.I. water in 25 mL methanol with 10 min stirring under ambient conditions. In addition to undoped ZnO, various amounts of aluminum trichloride were adopted as a dopant source and added into the same aqueous solution with nominal Al contents of 2.5 at%, 5 at% and 10 at%, respectively. The total deposition time of the as-deposited films was 45 min ± 5 min at 500 °C. Furthermore, the cleaning process of silica coated float glass substrate (15 cm × 4 cm × 3 mm) was conducted by successive washings utilizing D.I. water, acetone and isopropanol, respectively. Afterwards, the substrate was dried using compressed air to blow across the surface. Meanwhile, the graphite block was preheated up to 500 °C before the start of the reaction. The as-prepared aqueous solution was added into a Drechsel bottle and using a Vicks ultrasonic humidifier with operating frequency of 1.6 MHz an aerosol mist was generated, which was brought into the reaction chamber *via* a 1.5 L min^−1^ flow of N_2_. Once the precursor solution was exhausted, the nitrogen gas continued to pass through the reaction chamber until the substrate temperature dropped below 80 °C.

## Film characterization

The X-ray pattern of the films were measured by modified Bruker-Axs D8 diffractometer X-ray diffraction (XRD) with a detected angular range of 10° < 2*θ* < 66° and counted at 1 s per step (0.05° for each steps) under X-ray radiation of Cu kα1 (1.54056 Å) and Cu kα2 (1.54439 Å), respectively. Then the data was analysed by software MDI Jada 6 and identified peak positions were compared with a standard JCPDS database. Moreover, the surface morphology and film thicknesses were characterized by scanning electron microscopy (SEM) and Filmetrics F20 thin-film analyser instrument operating in reflectance mode calibrated against with AZO in ambience environment, respectively. Furthermore, the optical properties of the films such as total transmittance (*T*_total_) and diffuse transmittance (*T*_diffuse_) were conducted by using UV/Vis and near infrared region (NIR) spectrometer (Perkin Elmer Lambda 950) with air background detected at wavelength ranges of 320 to 2500 nm. Meanwhile, the haze factor was calculated by the equation: *f*_haze_ = *T*_diffuse_/*T*_total_. Additionally, the chemical constituents were identified by X-ray photoelectron spectroscopy (XPS) analysis and the data processing were performed by Casa XPS software with calibrated binding energy of adventitious carbon (284.5 eV). Finally, Hall effect measurements were employed to determine the electrical properties of the films such as free carrier concentration (*N*), Hall mobility (*μ*) and film resistivity (*ρ*), in which, a room temperature Ecopia HMS-3000 device was set up in van der Pauw configuration with current of 1 mA and permanent magnet field of 0.58 T.

Atomic Force Microscopy was performed using a Bruker Dimension Icon in non-contact Soft Tapping mode. Each scan was performed over a 5 × 5 μm area with a spatial resolution of 10 nm. The probe used to perform these scans was sourced from Bruker (NTESPA, MPP-11220-10) and had a nominal tip radius of 8 nm.

## Results and discussion

Thin films of ZnO and Al doped ZnO were prepared *via* the AACVD reaction of [Zn(O_2_CCH_3_)·2H_2_O] with acetylacetonate, deionized water and AlCl_3_ at a range of concentrations (0, 5 and 10 mol% relative to [Zn(O_2_CCH_3_)·2H_2_O]) in methanol. All films were well adhered to the glass substrates passing the Scotch tape test and resisted scratching by a stainless steel scalpel.^35^ The bulk Al concentration in films was measured using energy dispersive X-ray spectroscopy (EDS) to be 0, 2.9 and 6.1 at% relative to Zn for films grown using 0, 5 and 10 mol% AlCl_3_, respectively, therefore giving a AlCl_3_ relative incorporation precursor efficiency of *ca.* 60%.

**Fig. 1:**
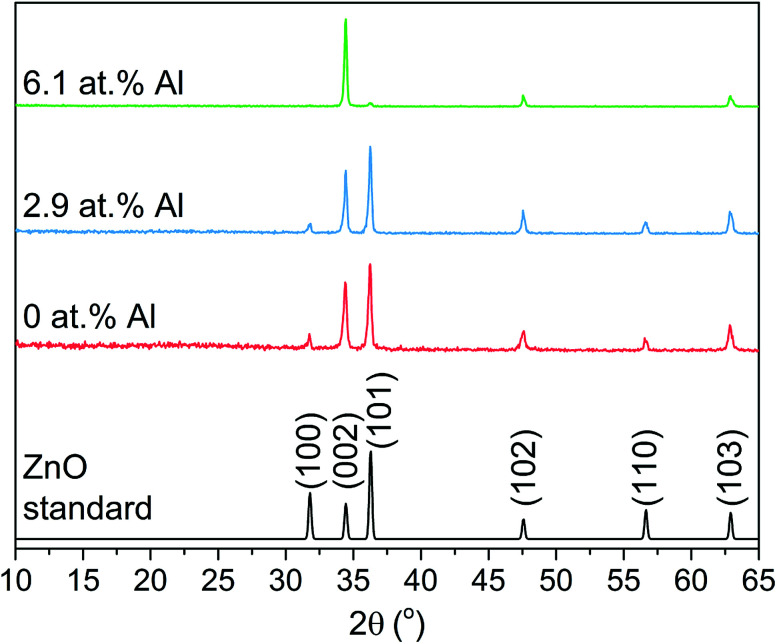
XRD pattern for the Al doped ZnO thin films grown *via* AACVD showing a good match to the hexagonal wurtzite standard ZnO.

X-ray diffraction (XRD) patterns in Fig. 1 of the as-prepared ZnO films correspond to the hexagonal wurtzite crystal structure (JCPDS 36-1451) with characteristic peaks at 31.7°, 34.4°, 36.2°, 47.5°, 56.5° and 62.8° that refer to the (100), (002), (101), (102), (110) and (212) planes, respectively. No peaks for any secondary oxide phases were observed, though it is important to note this does not rule out the presence of amorphous phases.

The XRD patterns were modeled to determine the ZnO unit cell parameters of the AACVD grown films (Table 1). The undoped film showed lattice constants that deviate from typical literature values owing to the strain caused on the ZnO lattice by film growth occurring on the amorphous substrate.^4^ Upon doping at 2.9 and 6.1 at% with Al, the unit cell volume contracted by 0.15 and 0.29%, respectively due to the smaller ionic radius of four coordinate Al^3+^ (0.39 Å) relative to Zn^2+^ (0.6 Å) for the same geometry.

**Table 1:** The unit cell parameters for the nominally undoped and Al doped ZnO films prepared by AACVD

Al conc./%	*a*/Å	*c*/Å	Volume/Å^3^	Contraction/%
0	3.2517(10)	5.2072(9)	47.68(6)	—
2.9	3.2508(7)	5.2053(5)	47.61(4)	0.15
6.1	3.2463(13)	5.2087(3)	47.54(1)	0.29

Relative to the standard ZnO pattern, the undoped and 2.9 at% Al doped AACVD grown films showed a preference for the [002] direction and a lack of growth in the [100] direction while all other peak intensities in the XRD pattern showed only minimal deviation. This is attributed to the strain caused to the ZnO unit cell due to film growth on amorphous SiO_2_ coated float glass substrates, which has been observed previously for both ZnO and other metal oxide systems.^4,36,37^ At a higher Al doping level of 6.1 at% further preference for the [002] direction was seen and this time with both the (100) and (101) planes almost completely suppressed. It is common for ZnO systems, irrespective of deposition technique and dopant, to have preference for the (002) plane as this has the lowest surface energy in the crystal.^38–43^ It ultimately results in growth along the *c*-axis perpendicular to the substrate, which is somewhat evident from imaging of the surface morphology.

**Fig. 2:**
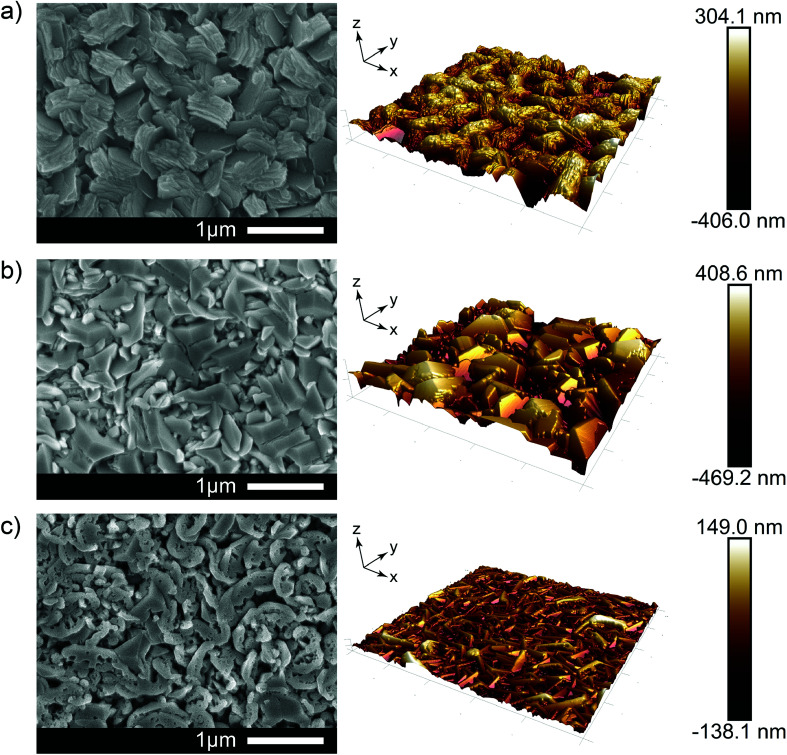
Shows the SEM and AFM images for (a) 0 at%, (b) 2.9 at% and (c) 6.1 at% doped ZnO thin films. AFM scans are presented of 5 × 5 μm regions for each sample and are projected at their true physical depths.

Scanning electron microscopy (SEM) and atomic force microscopy (AFM) images are shown in Fig. 2. For the undoped film, the morphology consists of many distinct sloping grains composed of numerous stacked hexagonal plates with an average diameter of ∼500 nm. This is a direct result of the acetylacetonate additive as normally ZnO films grown *via* AACVD using only [Zn(O_2_CCH_3_)·2H_2_O] and methanol at 500 °C (as was the case here) or below tend to give flat relatively featureless morphologies (see Fig. S1†). Keeping the amount of additive constant but doping to 2.9 at% with Al modifies the ZnO film further by giving not only interconnected large crystal grains (∼1 μm in diameter), but also widespread dense and uniform geometric configurations formed where many exposed triangular pyramidal shaped and wedge-like grains. At high levels of Al in the film, the morphology undergoes yet another change where many fragmentary small pieces of the grains and numerous pores on the crystal surface were observed. This is likely due to excessive impurities that interrupt continuity of crystal growth, instead, relatively unconsolidated and small pieces of grains inhibited side face growth in a hexagonal structure which agrees with the XRD results. It is noteworthy that these unique feature configurations were attributed to both the effects of dopants and additives, which indicates that adequate modification of additives or dopants could offer a promising method to alter the crystal growth pattern, thereby manipulating the physicochemical properties that the materials could achieve. Using either additives or dopant sources in many published work have presented limitations of further improvement of special characteristics in materials. The majority of textured ZnO research is focused on pre- or post-processing roughness features *via* additional etching processes rather than the formation of microstructures being integral to the primary oxide synthesis process. We obtained controlled wavelength-scale crystal growth and surface structures which are favorable as refraction sites for the purposes of light scattering in our TCOs.

**Fig. 3:**
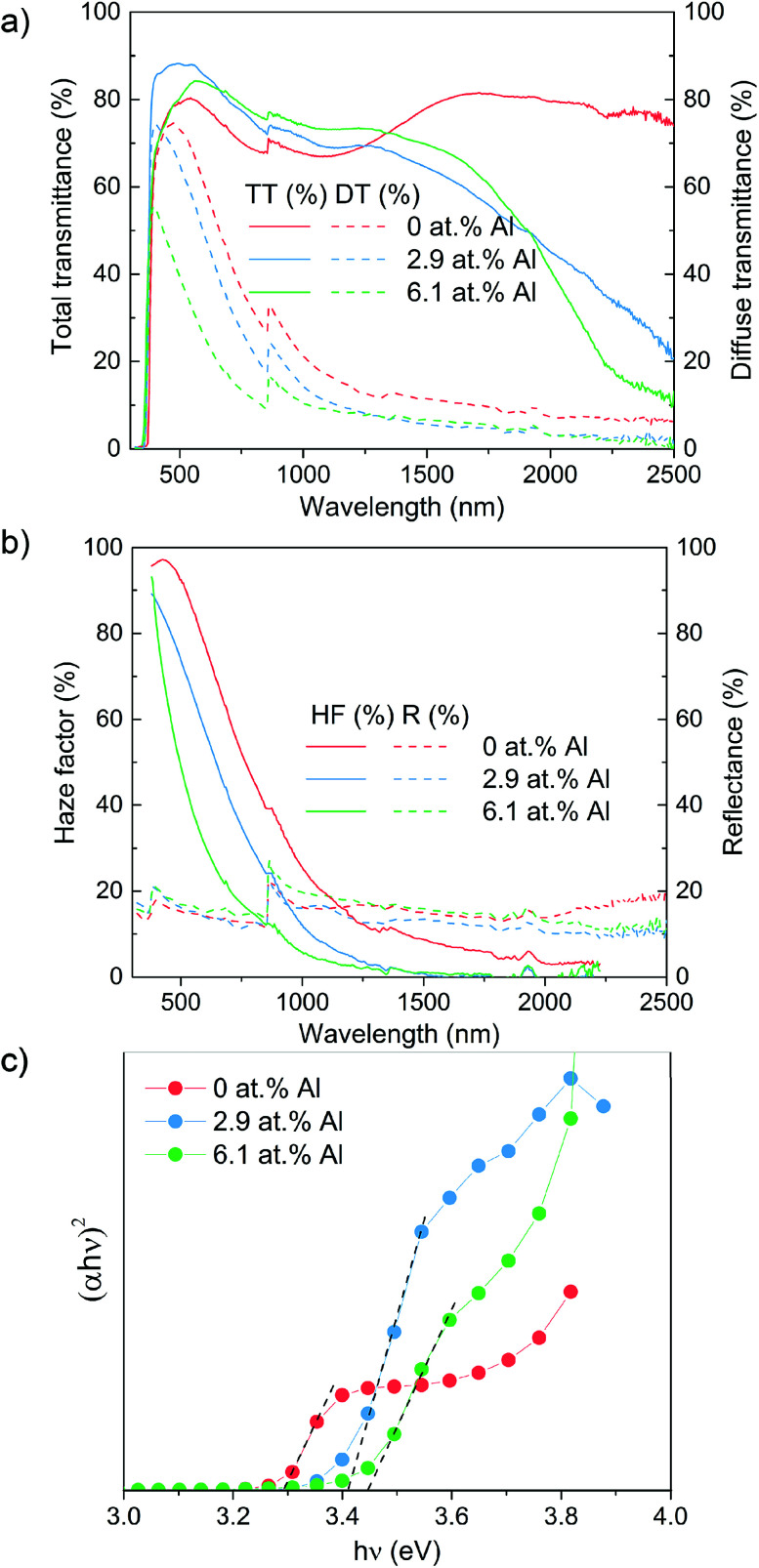
Optical results showing the (a) total transmittance/diffuse transmittance (b) haze factor/reflectance and (c) Tauc plot for the Al doped ZnO film grown using [Zn(O_2_CCH_3_)·2H_2_O] and [AlCl_3_].

The optical appearance of all as-prepared ZnO films including non-additive treated undoped ZnO film are shown in Fig. S2,† which visually indicate the optical properties changes because of synergistic effects of additive and dopant. In order to characterize the optical properties of the samples, total transmittance, diffuse transmittance and reflectance were measured by UV-Vis-NIR spectroscopy (Fig. 3). Across the visible wavelengths of 380–780 nm, both the doped films exhibit higher total transmittance (84% and 79% for 2.9% and 6.1 at% Al, respectively) than the undoped ZnO film and non-additive undoped ZnO film that had a transmittance of 74% and 60% (see Fig. S3†), respectively. The transmittance begins to decrease at wavelengths above 1500 nm for the doped films due to the increase in the free carrier concentration in the in the doped films (as shown from Hall effect data below). This reduction in transmittance was not observed for the undoped ZnO film. The diffuse transmittance, a measure of transmittance whereby light passing directly through the sample is not collected, was between 50–60% for all additive treated films across the visible wavelengths. While, the non-additive undoped film only has 0.7% diffuse transmittance in the same range of wavelengths (see Fig. S3†).

Using these two results in the ratio of *T*_diffuse_/*T*_total_, the haze factor was determined in an effort to study the light scattering capabilities of the films.^44^ The haze factor, averaged between 380 to 780 nm, can be tuned from 78% (undoped), to 63% (2.9% Al doped) and further to 40% (6.1% Al doping). The film with the highest Al concentration yielded lower roughness (35.9 nm RMS) films composed of small grains (approximately 700 × 50 nm). Such sub-wavelength structures facilitate an effective-medium interaction with light, which limits diffractive scattering. The characteristic grain sizes for undoped films (1000 × 400 nm) and high roughness (100 nm RMS) naturally lead to strong scattering in the visible regime, see Fig. 3. Comparing this scattering with that of films doped at 2.9% Al we observed a lower visible scattering despite grain sizes reaching up to 1400 nm in size and the film exhibiting a higher roughness (125 nm RMS). However, after taking into account the difference in thickness between these films measured by side-on SEM (920 nm undoped film; 700 nm 2.1% doped film) we find that scattering per unit of thickness actually increases for 2.9% Al concentrations. This thickness effect was also found to be true by Faÿ *et al.* who saw thicker B-doped ZnO films deposited *via* LPCVD to have higher haze due to the higher number of grains and therefore refraction sites.^45^ Therefore, a higher haze factor of the as-prepared samples could be expected as the thickness increase by prolonging the deposition time.

The 6.1% Al doped films in which haze is reduced, our understanding is that below a critical level, Al doping can lead to increased grain sizes and a strong and red-shifted scattering optical profile. However, doping beyond this point leads to smaller, sub-wavelength grains, passivated by an amorphous aluminium oxide, which reduce visible scattering through their sub-wavelength interaction with light.

Faÿ *et al.*'s B-doped ZnO films presented a lower haze value (<20%) at wavelength of 600 nm compared to our ZnO based films (77% for the undoped ZnO and 57% for the 2.9 at% Al doped sample) when films of similar thickness (800 to 900 nm) were compared. Although the Al doped ZnO samples exhibit relatively low haze values, the enhanced transmittance is more advantageous for transparent electrode applications. By comparison with literature, only few researches have shown high haze values over 60% across the visible spectrum. For instance, Kluth reported wet-chemical etching ZnO:Al with high haze value of 70% at 500 nm where the introduction of HCl solution was necessary to tailor the morphology of as-growth ZnO:Al films as a post-treatment process for highly textured ZnO.^7^ By contrast, the haze value at same wavelength in our studies were 93% and 75% for undoped ZnO and 2.9 at% ZnO:Al, respectively. The most representative work of pre-treatment textured AZO was presented by Hongsingthong, in which, they used carbon tetrafluoride as an etching gas to modify the morphology of the glass substrate, then a two-step MOCVD deposition was conducted by altering the precursors in two different layer deposition of ZnO films on the substrate. Consequently, the film indicated a very high haze value of 93% at wavelength of 550 nm (86% for undoped ZnO and 67% for 5 at% AZO in our studies).^46^ However, complicated preparation steps and volatile precursors were utilized which are of limited suitability for sustainable industrial applications.

The optical band gap of ZnO samples as determined by the Tauc plot reveals a regular shift from 3.30 eV for the undoped ZnO to 3.40 eV and 3.45 eV for the 2.9 and 6.1 at% Al doped films. This agrees well with literature examples and the shift in the band gap to higher values is explained by the Moss–Burstein effect involved with excess free electrons derived from Al^3+^ donor ions that occupy the bottom level of conduction band along with a rise of the Fermi level, thereby a higher excited energies required for electrons from a state to conduction band to above the Fermi level reflected in our work as an enlarged optical band gap.^37^ Thus, our studied films present excellent optical properties with competitive high haze values over 60% and 80% transmittance in the visible spectrum.

**Fig. 4:**
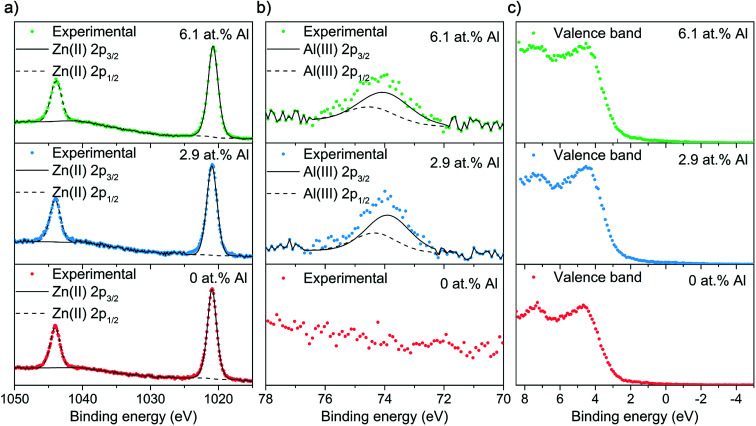
XPS spectra for the AACVD grown thin films showing the (a) Zn 2p, (b) Al 2p and (c) valence band transitions.

X-ray photoelectron spectroscopy (XPS) was employed to carry out surface specific compositional and oxidation state analysis (Fig. 4a and b). The Zn 2p transition for all films showed the typical symmetrical doublet separated by 23.2 eV with the 2p_3/2_ peaks appearing at 1021.0 eV and matching to literature values for Zn^2+^. For the doped films, the Al 2p peak showed a degree of asymmetry that was deconvoluted to a doublet with a peak separation of 0.41 eV. The Al 2p_3/2_ transition appears at 73.9 and 74.0 eV for the 2.9 and 6.1 at% Al, respectively and therefore corresponds to Al^3+^.

Fig. 4c shows the XPS valence band spectra. For all films, the peak corresponding to hybridized Zn 3d, Zn 4s and O 2p orbitals appears at 7.5 eV whereas the peak as a result of O 2p states mixed with Zn 3d and some Zn 4p orbitals is centered at 4 eV.^47–50^

**Fig. 5:**
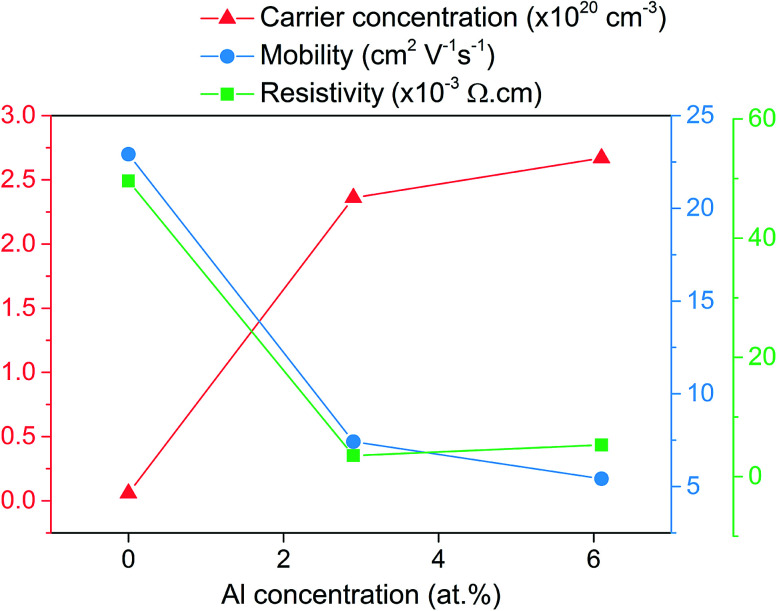
Hall effect results showing the change in carrier concentration, carrier mobility and resistivity upon doping of ZnO with Al.

The Hall effect measurements shown in Fig. 5 give insight into the electrical properties for the ZnO based films. The nominally undoped sample had a relatively high carrier concentration of 5.82 × 10^18^ cm^−3^ compared to what is usually reported in literature for pure ZnO films presumably due to adventitious hydrogen that resulted in a low resistivity of 4.96 × 10^−2^ Ω cm. The carrier concentration observed for the nominally undoped ZnO film here is even higher than what is typically seen for undoped ZnO films grown from [Zn(O_2_CCH_3_)·2H_2_O] *via* AACVD, therefore suggesting that the additive acetylacetonate may also be a source of adventitious hydrogen that is known to enhance the conductivity.

Upon doping to 2.9 at% with Al the carrier concentration increases by almost two orders of magnitude to 1.76 × 10^20^ cm^−3^ due to the release of up to one electron for every Zn^2+^ substituted by Al^3+^. The carrier mobility reduces from 22.92 cm^2^ V^−1^ s^−1^ that was observed for the undoped ZnO to 11.02 cm^2^ V^−1^ s^−1^ as a consequence of ionized impurity scattering that is know to be the major limiting factor in degenerately doped ZnO at carrier concentration between 10^20^ to 10^21^ cm^−3^.^51^ The resistivity for the 2.9 at% Al film was 3.54 × 10^−3^ Ω cm and the lowest of the three films. For the next film, despite the Al concentration being more than double at 6.1 at%, the carrier concentration only slightly increased to 2.67 × 10^20^ cm^−3^. This coupled with the fact that the carrier mobility also reduced to 5.42 cm^2^ V^−1^ s^−1^ while the resistivity increased to 5.31 × 10^−3^ Ω cm seems to indicate that in this sample either increased charge compensation (in the form of oxygen intestinals and zinc vacancies) is taking place and/or that some of the Al is in the form of electrically inactive Al_2_O_3._^1,52^

## Conclusion

In summary, for the first time, a single step synthesis route of using acetylacetone and D.I. water as additives in the preparation of ZnO thin films *via* AACVD was developed. The additives had a dramatic influence on the electrical and optical properties of as-deposited films, in which, the ultra large haze factor of 78% across the visible spectrum and competitive resistivity with high carrier mobility of 22.92 cm^2^ V^−1^ s^−1^ were achieved in undoped ZnO sample. Furthermore, the combination of Al dopant and additives into the precursor were successfully proved to enhance transmittance (≥83%), low resistivity (3.49 × 10^−3^ Ω cm) and high haze value (≥62%) in Al-doped ZnO samples which presents enormous potential for the application of front transparent electrode in Si based thin film solar cells with superior light scattering capability and potential cost effective for sustainable production.

## Conflicts of interest

The authors declare no conflict of interest.

## Supplementary Material

RA-008-C8RA09338E-s001
