# Supplementary material for: Single step route to highly transparent, conductive and hazy aluminium doped zinc oxide films
Source: RSC Adv. 2018 Dec 19;8(74):42300–7. doi: 10.1039/c8ra09338e (PMC9092156; doi:10.1039/c8ra09338e)
Supplement: RA-008-C8RA09338E-s001 [file RA-008-C8RA09338E-s001.pdf]

Electronic Supplementary Material (ESI) for RSC Advances.
This journal is © The Royal Society of Chemistry 2018

Single step route to highly transparent, conductive and hazy aluminium doped zinc oxide films

Jianwei Li[a], Sanjayan Sathasivam[a], Alaric Taylor[b], Claire J. Carmalt[a] and Ivan P. Parkin[a]*

*Corresponding author

[a]Materials Chemistry Centre, Department of Chemistry, University College London, 20 Gordon Street, London WC1H 0AJ, UK

[b] Department of Electronic & Electrical Engineering, University College London, Torrington Place, London WC1E 7JE, UK

Fax: (+44) 20-7679-7463

E-mail: i.p.parkin@ucl.ac.uk

Supplementary information:

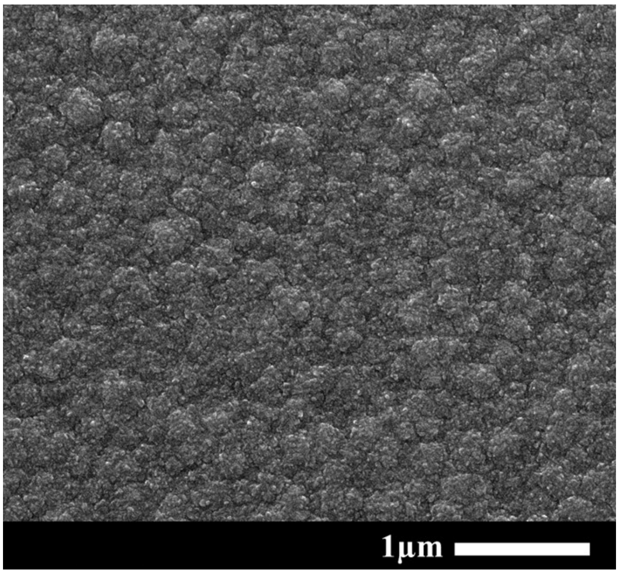

Figure S1: SEM image of as-prepared undoped ZnO thin film by AACVD under 500 °C without using acetylacetone as additive in the precursor.

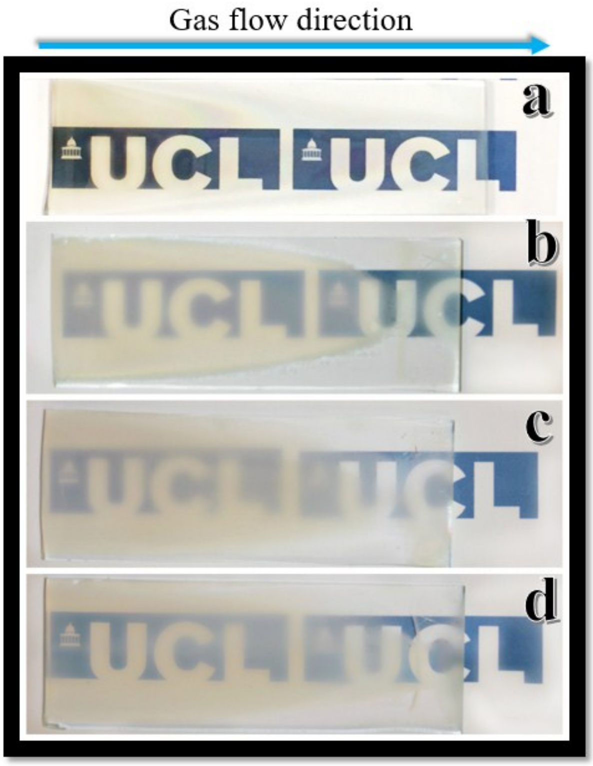

Figure S2: Digital photo showing the appearance of all as-prepared ZnO thin films： a) non-additive undoped ZnO film, b) additive treated undoped ZnO film, c) additive treated 2.9 at.% Al doped ZnO film and d) additive treated 6.1 at.% Al doped ZnO film.

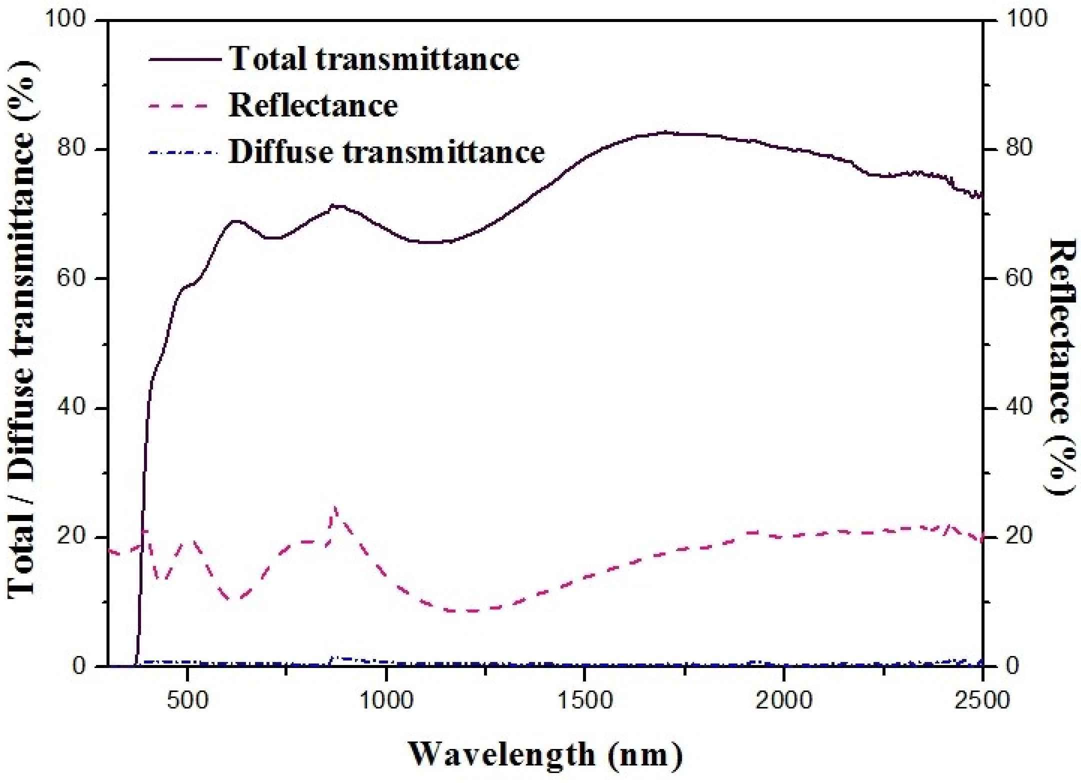

Figure S3: Optical properties of non-additive undoped ZnO film: Total transmittance, reflectance and diffuse transmittance.
